# Factors Influencing ICU Nurses’ Proficiency in Point‐of‐Care Gastrointestinal Ultrasound: A National Cross‐Sectional Study

**DOI:** 10.1155/jonm/3178958

**Published:** 2026-05-09

**Authors:** Gang Wang, Yingying Liu, Shengyun Wang, Silong Gao, Hui Lin, Qian Wu, Debin Huang, Yueshuai Pan, Yuxiao Zhao, Huimin Wei, Lili Wei

**Affiliations:** ^1^ Department of Critical Care Medicine, Affiliated Hospital of Qingdao University, Qingdao, China, qdu.edu.cn; ^2^ Organizational Management Department, Shandong Provincial Nursing Association, Jinan, China; ^3^ Liver Disease Center Intensive Care Unit, Affiliated Hospital of Qingdao University, Qingdao, China, qdu.edu.cn; ^4^ Department of Neonatal, Affiliated Hospital of Qingdao University, Qingdao, China, qdu.edu.cn; ^5^ Department of Critical Care Medicine, The First Affiliated Hospital of Guangxi Medical University, Nanning, China, gxmu.edu.cn; ^6^ Department of Nursing, Affiliated Hospital of Qingdao University, Qingdao, China, qdu.edu.cn; ^7^ Office of Dean, Affiliated Hospital of Qingdao University, Qingdao, China, qdu.edu.cn

**Keywords:** cross-sectional study, gastrointestinal ultrasound, influencing factors, intensive care unit, nurses

## Abstract

**Aims:**

To evaluate point‐of‐care gastrointestinal ultrasound (GI‐POCUS) proficiency, identify influencing factors, and examine its clinical application among intensive care unit (ICU) nurses in China.

**Background:**

GI‐POCUS supports gastrointestinal function assessment, enteral nutrition guidance, and dysfunction detection in patients who are critically ill. However, its use among ICU nurses remains limited, and the factors influencing proficiency are unclear.

**Methods:**

A national cross‐sectional survey was conducted among 2009 ICU nurses from 21 provinces in China using a structured online questionnaire covering their demographic and unit characteristics, GI‐POCUS training, practice, skills, proficiency, and clinical application. Participants were recruited from various ICU settings, with the majority from adult ICUs and a small proportion from pediatric and neonatal ICUs. Data were analyzed using SPSS 27.0 with descriptive statistics, chi‐square tests, *t*‐tests, and multivariate logistic regression.

**Results:**

Only 297/2009 (14.8%) of ICU nurses rated their GI‐POCUS proficiency as “good,” whereas 1205/2009 (59.9%) rated it as “poor.” GI‐POCUS proficiency was significantly associated with billability, training participation, usage frequency, documentation of GI‐POCUS findings in nursing charts, proficiency in probe operations, ability to perform qualitative and quantitative analyses, and diagnostic accuracy and sensitivity (*p* < 0.05). Clinically, only 492/2009 (24.5%) of ICU nurses could identify intestinal necrosis or ischemia. 1232/2009 (61.3%) applied GI‐POCUS to assess gastric emptying and residual volume, and 1069/2009 (53.2%) used it to guide feeding tube placement. 1032/2009 (51.4%) of ICU nurses could identify gastric transverse and longitudinal section images, whereas the recognition rate for intestinal wall blood flow signals was the lowest at only 369/2009 (18.4%).

**Conclusion:**

GI‐POCUS proficiency among ICU nurses in China is low. Promoting sustainable implementation requires organizational support, leadership engagement, and integration into routine critical care nursing practice.

**Implications for Leaders:**

Nursing leaders should establish structured GI‐POCUS training pathways, integrate ultrasound use into routine ICU workflows and electronic documentation systems, and advocate for institutional recognition including billing mechanisms. These leadership actions can enhance nurses’ proficiency and promote sustainable nurse‐led GI‐POCUS implementation.

## 1. Introduction

Point‐of‐care ultrasound (POCUS) is increasingly recognized as a real‐time, noninvasive, and repeatable imaging tool with growing importance in critical care medicine [1–3]. Unlike conventional imaging, POCUS is performed by healthcare workers at the bedside during patient management, which significantly reduces the interval from diagnosis to intervention and provides immediate information to guide physiological assessment and therapeutic decision‐making [4, 5]. With advances in portable ultrasound devices and the development of simplified scanning protocols, POCUS is no longer confined to physicians or radiologists. Instead, it is progressively being integrated into nursing practice, particularly in intensive care units (ICUs), where nurses play a central role in continuous patient monitoring and timely clinical responses [1, 6–9].

Gastrointestinal (GI) function plays a critical role in the outcome of patients who are critically ill. GI dysfunction not only compromises nutritional support but is also associated with increased intestinal permeability, higher infection risk, and poorer prognosis [10, 11]. Early identification and dynamic monitoring of GI function are therefore essential components of high‐quality critical care nursing. However, traditional methods for assessing GI function, such as gastric tube aspiration, abdominal radiography, computed tomography, and gastric emptying tests, are limited by their invasiveness, procedural delay, and difficulty in frequent repetition [12]. In this context, point‐of‐care gastrointestinal ultrasound (GI‐POCUS) has emerged as a promising tool that allows noninvasive, repeatable, and dynamic assessment of gastric and intestinal function at the bedside [13, 14]. This technique enables the assessment of various GI physiological parameters, including gastric residual volume, gastric emptying, intestinal peristalsis, luminal diameter changes, and intra‐abdominal fluid status, providing valuable imaging evidence to guide enteral nutrition strategies and early detection of GI dysfunction in patients who are critically ill [9, 13]. Growing evidence suggests that GI‐POCUS demonstrates good reproducibility and reliability for measuring gastric residual volume and gastric emptying, with potential advantages over traditional aspiration‐based methods [9, 14]. Moreover, the utility of GI‐POCUS for monitoring intestinal distension, ascites, bowel wall edema, and blood flow alterations has been actively investigated [13].

Unlike many other POCUS applications that primarily support medical diagnosis, GI‐POCUS is closely aligned with nurses’ routine responsibilities in ICU care. From a nursing practice perspective, GI‐POCUS mainly supports four key care domains: assessment of feeding tolerance and gastric emptying, estimation of gastric residual volume, bedside guidance of feeding tube placement, and evaluation of bowel perfusion and ischemic risk [15, 16]. These domains are highly nurse‐led, time‐sensitive, and embedded within daily ICU workflows, particularly in enteral nutrition management and early identification of GI complications. Consequently, nurses’ competency in GI‐POCUS significantly influences feeding safety, continuity of nutritional support, and the early recognition of GI complications in critically ill patients. At the same time, these characteristics make GI‐POCUS especially vulnerable to variability in training opportunities, workload pressures, and organizational support, highlighting the critical role of nursing leadership in sustaining competency and ensuring consistent practice.

Despite its emerging clinical value, and strong alignment with nursing practice, the integration of GI‐POCUS into routine nursing practice remains challenging. Historically, ultrasound training has focused on physicians, and nurses’ access to structured education, supervised practice, and formal competency assessment has been limited [2]. As a result, GI‐POCUS implementation in nursing practice is often fragmented and informal, relying heavily on local nursing leadership support rather than standardized organizational strategies. From a nursing management perspective, the successful adoption of GI‐POCUS extends beyond individual skill acquisition and reflects broader organizational factors, including training structures, workflow integration, documentation systems, quality assurance mechanisms, and institutional recognition of nursing‐led ultrasound practice [17]. Without clear governance frameworks, standardized operating procedures (SOPs), and sustained leadership support, nurses may perform GI‐POCUS inconsistently, limiting its clinical impact and long‐term sustainability.

Although interest in nurse‐performed GI‐POCUS is increasing, empirical evidence regarding ICU nurses’ proficiency and its influencing factors remains limited, particularly large‐scale, multicenter surveys [9]. Given the pivotal role of nurses in the daily management, nutritional support, and complication monitoring of patients who are critically ill, gaining an in‐depth understanding of Chinese ICU nurses’ proficiency in GI‐POCUS, analyzing its influencing factors, and assessing its clinical application is crucial for identifying key barriers to technology adoption and informing leadership strategies. Therefore, this study aimed to investigate the perceived proficiency level, influencing factors, and current clinical application status of GI‐POCUS among Chinese ICU nurses through a national questionnaire‐based survey. By doing so, this study seeks to provide evidence‐based guidance for nursing leaders and managers in designing targeted training programs and optimizing clinical workflows to support the ongoing competency of nurses engaging with GI‐POCUS and enhance its safe and effective integration into critical care nursing practice.

## 2. Methods

### 2.1. Design

A multicenter, cross‐sectional study was conducted.

### 2.2. Sample

This study recruited ICU nurses from 21 provinces across China between September and October 2025 using a convenience sampling approach. The inclusion criteria were as follows: (a) ≥ 1 year of ICU work experience, (b) possessing a valid nurse practicing certificate, (c) voluntary participation with signed informed consent, (d) clinical experience in applying GI‐POCUS, and (e) ability to complete the online questionnaire independently. Nonclinical nursing staff were excluded from the study.

The sample size was calculated based on 27 variables, including 9 related to demographic information, 6 to department information, 11 to GI‐POCUS training, practice, and skills, and 1 to GI‐POCUS proficiency level. According to the Kendall estimation method, the recommended sample size should be five to 10 times the number of variables [18]. Allowing for a potential 20% rate of invalid responses, the required sample size was estimated to range from 162 to 324 participants. Informed consent was included in the preamble of the questionnaire, and all participants voluntarily provided informed consent.

Participants were recruited from various ICU settings, with the majority working in adult ICUs and a small proportion from pediatric and neonatal ICUs. Due to the limited number of pediatric and neonatal ICU nurses, no separate subgroup analysis was conducted.

### 2.3. Procedure

The research team liaised with ICU directors from participating hospitals to distribute the survey using Wenjuanxing (https://www.wjx.cn), the largest online survey platform in China. The preface of the questionnaire provided detailed information regarding the study objectives, the researcher’s identities, the significance of the research, and informed consent. To ensure data quality and completeness, the electronic questionnaire was designed with built‐in logical constraints that allowed each respondent to submit only one response and required all items to be completed before submission.

### 2.4. Instruments

The research team developed an initial questionnaire based on a comprehensive literature review. The draft was subsequently reviewed and revised by a panel of five experts in critical care nursing and ultrasonography, resulting in a preliminary version. A pilot test was conducted with 30 ICU nurses to ensure the clarity and feasibility of the questionnaire items. Feedback from this pilot phase was incorporated, and necessary revisions were made to finalize the instrument.

The questionnaire consisted of five sections: (1) demographic information of participants; (2) basic information of the department; (3) GI‐POCUS training, practice, and skills; (4) GI‐POCUS proficiency level; and (5) clinical application of GI‐POCUS. Nurses’ perceived proficiency levels in GI‐POCUS were self‐assessed and categorized into three levels: (a) Good: It demonstrates solid theoretical knowledge and practical skills in GI‐POCUS; independently performs procedures such as gastric residual volume monitoring, gastric motility assessment, and feeding tube placement verification with sound clinical judgment; and participates in teaching, training, or research activities. (b) General: It possesses basic theoretical knowledge and operational skills in GI‐POCUS and can perform certain tasks under guidance but has limited ability to handle complex cases or make comprehensive clinical judgments. (c) Poor: It has a limited understanding of the basic concepts, principles, and operational techniques of GI‐POCUS, with minimal independent practical experiences. In this study, Cronbach’s α for the questionnaire was 0.829, indicating good internal consistency.

Scoring procedures: The questionnaire did not generate a single total score. Each item was analyzed as an independent categorical or continuous variable. The self‐assessed GI‐POCUS proficiency level (three levels: “poor,” “general,” and “good”) was coded as 1 = “*poor,*” 2 = “*general,*” and 3 = “*good,*” and used as the outcome variable in regression analyses. Binary items (e.g., “Yes/No”) were coded as 0 = “*No*” and 1 = “*Yes.*” Nominal variables with more than two categories (e.g., frequency of use: “low,” “relatively low,” and “high”) were transformed into dummy variables, with the lowest category serving as the reference. Continuous variables (e.g., age, years of experience, and number of ultrasound equipment) were analyzed as raw values. No reverse scoring or composite scoring calculation was applied.

### 2.5. Statistical Analysis

Data were analyzed using the SPSS software (Version 27.0). Continuous variables with normal distribution, such as age, were presented as mean ± standard deviation (*M* ± SD), while categorical variables such as gender, professional title, and position were expressed as frequencies and percentages. Comparisons between two groups were performed using the independent‐samples *t*‐test or Mann–Whitney *U* test. For comparisons among multiple groups, data normality was first assessed using the Shapiro–Wilk test, and homogeneity of variances was evaluated using Levene’s test. When the data met the assumptions of normality and homogeneity of variance, one‐way analysis of variance (ANOVA) was conducted; otherwise, the Kruskal–Wallis nonparametric test was applied. Group comparisons for categorical variables were performed using the *χ* ^2^ test or Fisher’s exact test as appropriate. To account for multiple comparisons across all univariate analyses, the Benjamini–Hochberg false discovery rate (FDR) procedure was applied. An FDR‐adjusted *p* value < 0.05 was considered statistically significant.

Variables that remained significant after the FDR correction were included in a multinomial logistic regression model to identify the factors associated with different levels of GI‐POCUS proficiency (good, moderate, and poor). For the multivariate logistic regression model (a single prespecified model), no additional multiple comparison correction was applied, and a *p* value < 0.05 was considered significant.

### 2.6. Ethical Considerations

Ethical approval for this study was obtained from the ethics review committee of the Affiliated Hospital of Qingdao University (approval No. QYFYWZLL30728). Following approval from all participating hospitals, online questionnaires and informed consent forms were distributed to the potential participants. Their agreement to participate was asserted by choosing the “I agree” option ahead of filling out the questionnaires, which ensured that all respondents fully agreed to participate. Participation was entirely anonymous, and respondents were informed that all personal data would remain strictly confidential. This study was reported in accordance with the Checklist for Reporting Results of Internet E‐Surveys (CHERRIES).

## 3. Results

A total of 2137 questionnaires were returned. After excluding incomplete or invalid responses, 2009 valid questionnaires were included in the final analysis, yielding an effective response rate of 2009/2137 (94.0%). The final sample comprised ICU nurses from 21 provinces and cities who used GI‐POCUS technology. The participants were mainly female (76.0%), with an average age of (33.07 ± 6.00) years, and the working years and ICU working years were (10.33 ± 6.10) years and (7.84 ± 5.25) years, respectively.

For univariate analyses, chi‐square (*χ* ^2^) tests were used for categorical variables and one‐way ANOVA for continuous variables. To control for multiple comparisons, the Benjamini–Hochberg FDR procedure was applied. The original *p* values are presented in Tables 1, 2, 3, and all variables described as significant below remained significant after FDR correction (adjusted *p* < 0.05).

**TABLE 1:** Demographic information of participants and univariate analysis of GI‐POCUS proficiency among ICU nurses with different characteristics.

Characteristics	*N* (*n* = 2009) *n* (%)	Good (*n* = 297) *n* (%)	General (*n* = 508) *n* (%)	Poor (*n* = 1204) *n* (%)	*χ* ^2^ */F*	*p*
Age (years, *M* ± SD)	33.07 ± 6.00	31.70 ± 5.70	32.72 ± 5.95	33.55 ± 6.03	12.535^2^	< 0.001
Gender					30.586^1^	< 0.001
Female	482 (24.0)	91 (18.9)	154 (32.0)	237 (49.2)		
Male	1527 (76.0)	206 (13.5)	354 (23.2)	967 (63.3)		
Education					17.519^1^	0.002
Junior college	90 (4.5)	26 (28.9)	25 (27.8)	39 (43.3)		
Undergraduate	1866 (92.9)	264 (14.1)	471 (25.2)	1131 (60.6)		
Masters or above	53 (2.6)	7 (13.2)	12 (22.6)	34 (64.2)		
Professional title					29.134^1^	< 0.001
Primary level	869 (43.3)	161 (18.5)	234 (26.9)	474 (54.5)		
Intermediate level	1016 (50.6)	129 (12.7)	237 (23.3)	650 (64.0)		
Advanced level	124 (6.2)	7 (5.6)	37 (29.8)	80 (64.5)		
Job title					12.236^1^	0.002
No	1827 (90.9)	286 (15.7)	459 (25.1)	1082 (59.2)		
Head nurse/director	182 (9.1)	11 (6.0)	49 (26.9)	122 (67.0)		
Years of work experience (years, *M* ± SD)	10.33 ± 6.10	9.01 ± 5.48	9.95 ± 6.05	10.81 ± 6.22	11.78^2^	< 0.001
Years of ICU experience (years, *M* ± SD)	7.84 ± 5.25	6.71 ± 5.17	7.96 ± 5.37	8.07 ± 5.18	8.200^2^	< 0.001
Hospital grade					0.317^1^	0.853
Secondary	239 (11.9)	38 (15.9)	61 (25.5)	140 (58.6)		
Tertiary	1770 (88.1)	259 (14.6)	447 (25.3)	1064 (60.1)		
Categories of ICU					16.803^1^	0.078
General	1436 (71.5)	212.3 (14.8)	384 (26.7)	839 (58.4)		
Medical	92 (4.6)	12 (13.0)	20 (21.7)	60 (65.2)		
Surgical	210 (10.5)	34 (16.2)	53 (25.2)	123 (58.6)		
Emergency/trauma	175 (8.7)	29 (16.6)	35 (20.0)	111 (63.4)		
Pediatric/neonatal	50 (2.5)	4 (8.0)	5 (10.0)	41 (82.0)		
Other	46 (2.3)	5 (10.9)	11 (23.9)	30 (65.2)		

*Note:* Benjamini–Hochberg correction for multiple comparisons was applied; statistical significance remained unchanged.

^1^ *χ* ^2^ test.

^2^ANOVA.

**TABLE 2:** Department of information and univariate analysis of GI‐POCUS proficiency.

Characteristics	*N* (*n* = 2009) *n* (%)	Good (*n* = 297) *n* (%)	General (*n* = 508) *n* (%)	Poor (*n* = 1204) *n* (%)	*χ* ^2^ */F*	*p*
Nurse–patient ratio					2.166^1^	0.705
Below 1:2	449 (22.3)	63 (14.0)	108 (24.1)	278 (61.9)		
1:2 to 1:3	1134 (56.4)	167 (14.7)	299 (26.4)	668 (58.9)		
More than 1:3	426 (21.2)	67 (15.7)	101 (23.7)	258 (60.6)		
Whether nurses were allowed to perform GI‐POCUS independently					54.768^1^	< 0.001
No	370 (18.4)	32 (8.6)	55 (14.9)	283 (76.5)		
Yes	1639 (81.6)	265 (16.2)	453 (27.6)	921 (56.2)		
Whether to incorporate GI‐POCUS into routine daily assessments					198.794^1^	< 0.001
No	1737 (86.5)	187 (10.8)	422 (24.3)	1128 (64.9)		
Yes	272 (13.5)	110 (40.4)	86 (31.6)	76 (27.9)		
Whether GI‐POCUS examinations were billable					149.222^1^	< 0.001
No	1696 (84.4)	187 (11.0)	412 (24.3)	1097 (64.7)		
Yes	313 (15.6)	110 (35.1)	96 (30.7)	107 (34.2)		
Whether to establish a standardized operating procedure for GI‐POCUS					225.561^1^	< 0.001
No	1671 (83.2)	173 (10.4)	385 (23.0)	1113 (66.6)		
Yes	338 (16.8)	124 (36.7)	123 (36.4)	91 (26.9)		
Whether to establish a GI‐POCUS quality control system					253.657^1^	< 0.001
No	1347 (67.0)	112 (8.3)	270 (20.0)	965 (71.6)		
Yes	662 (33.0)	185 (27.9)	238 (36.0)	239 (36.1)		
Whether to establish a GI‐POCUS mentorship system					266.145^1^	< 0.001
No	1400 (69.7)	118 (8.4)	286 (20.4)	996 (71.7)		
Yes	609 (30.3)	179 (29.4)	222 (36.5)	208 (34.2)		
Number of ultrasound equipment (*M* ± SD)	1.50 ± 1.12	1.56 ± 1.35	1.67 ± 1.22	1.41 ± 1.01	10.109^2^	< 0.001

*Note:* Benjamini–Hochberg correction for multiple comparisons was applied; statistical significance remained unchanged.

^1^ *χ* ^2^ test.

^2^ANOVA.

**TABLE 3:** GI‐POCUS training, practice, and skills of participants and univariate analysis of GI‐POCUS proficiency.

Characteristics	*N* (*n* = 2009) *n* (%)	Good (*n* = 297) *n* (%)	General (*n* = 508) *n* (%)	Poor (*n* = 1204) *n* (%)	*χ* ^2^	*p*
Whether you have received training					286.694	< 0.001
No	1098 (54.7)	89 (8.1)	166 (15.1)	843 (76.8)		
Yes	911 (45.3)	208 (22.8)	342 (37.5)	361 (39.6)		
Duration of GI‐POCUS use					177.143	< 0.001
< 3 months	1428 (71.1)	175 (12.3)	270 (18.9)	983 (68.8)		
3 months–2 years	367 (18.3)	75 (20.4)	167 (45.5)	125 (34.1)		
> 2 years	214 (10.7)	47 (22.0)	71 (33.2)	96 (44.9)		
Frequency of GI‐POCUS use					427.070	< 0.001
Low	1523 (75.8)	143 (9.4%)	284 (18.6%)	1096 (72.0)		
Relatively low	406 (20.2)	110 (27.1%)	193 (47.5%)	103 (25.4%)		
High	80 (4.0)	44 (55.0%)	31 (38.8%)	5 (6.3%)		
Whether nursing care plans are adjusted based on GI‐POCUS results					221.616	< 0.001
No	1173 (57.4)	78 (6.6)	245 (20.9)	850 (72.5)		
Yes	836 (41.6)	219 (26.2)	263 (31.5)	354 (42.3)		
Acceptance of GI‐POCUS–based nursing plan adjustments by attending physicians					171.870	< 0.001
Often	729 (36.3)	188 (25.8)	181 (24.8)	360 (49.4)		
Occasionally	668 (33.3)	69 (10.3)	222 (33.2)	377 (56.4)		
Rarely/Not at all	612 (30.5)	40 (6.5)	105 (17.2)	467 (76.3)		
Whether GI‐POCUS results are recorded in nursing charts					275.387	< 0.001
No	1305 (65.0)	82 (6.3)	292 (22.4)	931 (71.3)		
Yes	704 (35.0)	215 (30.5)	216 (30.7)	273 (38.8)		
Proficiency in probe operation					377.439	< 0.001
No	1653 (82.2)	150 (9.1)	361 (21.9)	1141 (69.1)		
Yes	357 (17.8)	147 (41.2)	147 (41.2)	63 (17.6)		
Whether qualitative and quantitative analyses can be performed					415.989	< 0.001
No	1585 (78.9)	137 (8.6%)	323 (20.4)	1125 (71.0)		
Yes	424 (21.1)	160 (37.7%)	185 (43.6)	79 (18.6)		
Perceived diagnostic accuracy of GI‐POCUS					385.906	< 0.001
Good	636 (31.7)	196 (30.8%)	144 (22.6)	296 (46.5)		
General	790 (39.3)	71 (9.0%)	312 (39.5)	407 (51.5)		
Poor	583 (29.0)	30 (5.1%)	52 (8.9)	501 (85.9)		
Perceived diagnostic sensitivity of GI‐POCUS					401.378	< 0.001
Good	654 (32.6)	204 (31.2%)	150 (22.9)	300 (45.9)		
General	801 (39.9)	68 (8.5%)	312 (39.0)	421 (52.6)		
Poor	554 (27.6)	25 (4.5%)	46 (8.3)	483 (87.2)		

*Note:* Benjamini–Hochberg correction for multiple comparisons was applied; statistical significance remained unchanged.

### 3.1. Demographic Information of Participants and Univariate Analysis of GI‐POCUS Proficiency Among ICU Nurses With Different Characteristics

As shown in Table 1, the following demographic variables were significantly associated with GI‐POCUS proficiency: age, gender, education, professional title, job title, total work experience, and ICU work experience (all *p* < 0.05). Nurses reporting “good” proficiency were younger and had fewer years of total and ICU experience than those reporting “poor” proficiency. A higher proportion of male nurses and those with a bachelor’s degree or higher reported “good” proficiency. Hospital grade and ICU category were not significantly associated with proficiency.

### 3.2. Department of Information and Univariate Analysis of GI‐POCUS Proficiency

Several department‐level factors were significantly associated with GI‐POCUS proficiency, including permission to perform GI‐POCUS independently, incorporation into routine daily assessments, billability, presence of SOPs, quality control systems, mentorship systems, and number of ultrasound devices (all *p* < 0.05). Nurses working in departments with billable GI‐POCUS examinations or mentorship systems reported higher levels of proficiency. Nurse–patient ratio was not significantly associated with proficiency (Table 2).

### 3.3. GI‐POCUS Training, Practice, and Skills of Participants and Univariate Analysis of GI‐POCUS Proficiency

All examined training, practice, and skills‐related variables were significantly associated with GI‐POCUS proficiency (all *p* < 0.001). Nurses, who had received GI‐POCUS training, used the technique more frequently, documented the findings in nursing charts, and reported higher proficiency in probe operation, and qualitative/quantitative analyses were more likely to report better proficiency levels. Perceived diagnostic accuracy and sensitivity were also significantly associated with proficiency (Table 3).

### 3.4. Multivariate Analysis of Participants’ GI‐POCUS Proficiency Level

To identify independent predictors of ICU nurses’ GI‐POCUS proficiency, a multinomial logistic regression analysis was conducted using self‐rated proficiency (poor, general, and good) as the outcome variable. Variables that remained statistically significant after FDR correction in univariate analyses were entered into the model. The results are presented in Table 4, with odds ratios (ORs) and 95% confidence intervals (CIs).

**TABLE 4:** Multivariate analysis of participants’ GI‐POCUS proficiency level.

Characteristics	Good vs. poor^1^			General vs. poor^1^			General vs. good^2^		
	*p*	OR	95% CI	*p*	OR	95% CI	*p*	OR	95% CI
Whether GI‐POCUS examinations were billable (Yes)									
No	< 0.001	0.456	0.296–0.702	0.052	0.688	0.471–1.004	0.044	1.508	1.012–2.247
Whether you have received training (Yes)									
No	0.119	0.733	0.496–1.083	< 0.001	0.556	0.419–0.737	0.189	0.759	0.502–1.145
Frequency of GI‐POCUS use (High)									
Low	< 0.001	0.047	0.016–0.142	< 0.001	0.168	0.061–0.466	< 0.001	3.542	1.768–7.095
Relatively low	< 0.001	0.126	0.042–0.377	0.063	0.378	0.135–1.054	< 0.001	2.992	1.560–5.740
Whether GI‐POCUS results are recorded in nursing charts (Yes)									
No	< 0.001	0.347	0.231–0.521	0.012	0.666	0.486–0.913	0.002	1.917	1.271–2.893
Proficiency in probe operation (Yes)									
No	< 0.001	0.332	0.195–0.564	0.046	0.640	0.413–0.992	0.007	1.930	1.195–3.116
Whether qualitative and quantitative analysis can be performed (Yes)									
No	0.106	0.652	0.388–1.095	< 0.001	0.417	0.279–0.624	0.076	0.640	0.390–1.049
Perceived diagnostic accuracy of GI‐POCUS (Poor)									
General	0.893	0.945	0.413–2.164	0.014	2.063	1.156–3.681	0.085	2.184	0.899–5.307
Perceived diagnostic sensitivity of GI‐POCUS (Poor)									
Good	0.005	4.227	1.555–11.489	0.100	1.863	0.887–3.910	0.132	0.441	0.152–1.281
General	0.162	1.862	0.779–4.449	0.004	2.396	1.311–4.377	0.599	1.287	0.502–3.296

^1^Using “Poor proficiency” as the reference group.

^2^Using “Good proficiency” as the reference group.

Compared with nurses reporting “poor” proficiency, those who used GI‐POCUS more frequently were significantly more likely to report “good” proficiency. The OR for low‐frequency users versus high‐frequency users was 0.047 (95% CI: 0.016–0.142, *p* < 0.001), indicating substantially lower odds of “good” proficiency among low‐frequency users. Similarly, nurses who documented GI‐POCUS findings in nursing charts were more likely to report “good” rather than “poor” proficiency (OR for nondocumenters vs. documenters = 0.347, 95% CI: 0.231–0.521, *p* < 0.001). Proficiency in probe operation also independently distinguished “good” from “poor” proficiency, with an OR of 0.332 (95% CI: 0.195–0.564, *p* < 0.001) for nonproficient versus proficient nurses.

Organizational factors also played a significant role. Nurses working in departments where GI‐POCUS examinations were billable were more likely to report “good” proficiency than those in nonbillable settings (OR for nonbillable vs. billable = 0.456, 95% CI: 0.296–0.702, *p* < 0.001). In contrast, training participation was associated with the transition from “poor” to “general” proficiency (OR for untrained vs. trained = 0.556, 95% CI: 0.419–0.737, *p* < 0.001) but did not significantly predict “good” proficiency.

Regarding diagnostic skills, nurses who perceived higher diagnostic sensitivity were significantly more likely to belong to the “good” proficiency group than the “poor” group (OR = 4.227, 95% CI: 1.555–11.489, *p* = 0.005). Ability to perform qualitative and quantitative analyses distinguished “general” from “poor” proficiency (OR for unable vs. able = 0.417, 95% CI: 0.279–0.624, *p* < 0.001) but did not significantly differentiate “good” from “poor”. Nurses who perceived their diagnostic accuracy as “general” (vs. “poor”) were more likely to have “general” rather than “poor” proficiency (OR = 2.063, 95% CI: 1.156–3.681, *p* = 0.014), but this factor did not significantly differentiate “good” from “poor” proficiency (Table 4).

### 3.5. Status of GI‐POCUS Clinical Application Among Participants

**FIGURE 1:**
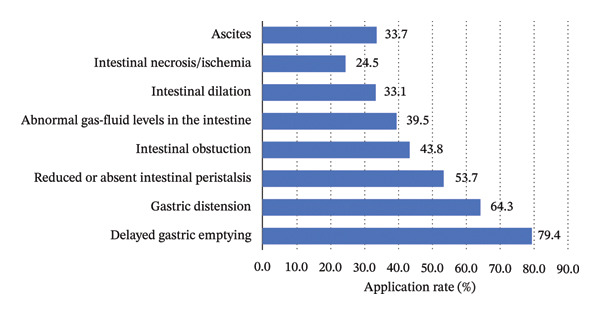
Gastrointestinal dysfunctions that participants were able to identify using GI‐POCUS.

As shown in Figure 1, the most frequently identified GI dysfunction was delayed gastric emptying (79.4%). In contrast, the ability to identify intestinal necrosis or ischemia was notably low (24.5%), indicating a gap in recognizing complex pathologies.

**FIGURE 2:**
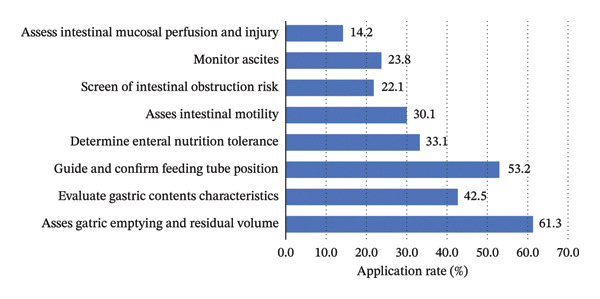
Clinical scenarios in which participants apply GI‐POCUS.

Regarding clinical scenarios (Figure 2), the most common application of GI‐POCUS was assessing gastric emptying or residual volume (61.3%), followed by guiding feeding tube placement (53.2%). Less frequent applications included screening for intestinal obstruction (22.1%) and evaluating intestinal mucosal perfusion and injury (14.2%), suggesting that nurses predominantly use GI‐POCUS for routine enteral nutrition management rather than advanced diagnostic assessments.

**FIGURE 3:**
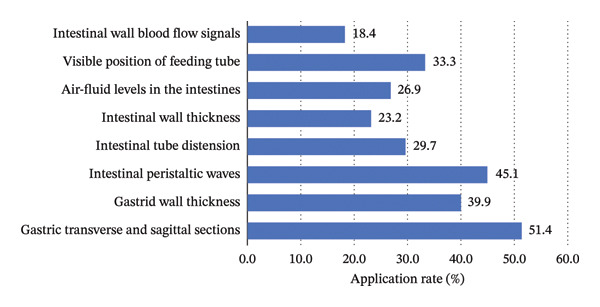
GI‐POCUS images that participants were able to interpret accurately.

In terms of image interpretation (Figure 3), more than half of the nurses could accurately identify gastric transverse and longitudinal sections (51.4%). However, recognition rates for pathological findings were substantially lower: gas‐fluid levels (26.9%), intestinal wall thickening (23.2%), and intestinal wall blood flow signals (18.4%).

## 4. Discussion

This cross‐sectional survey evaluated Chinese ICU nurses’ GI‐POCUS proficiency, identified influencing factors, and examined its current clinical application. From a nursing management perspective, these findings not only reflect individual nurses’ technical capabilities but also reveal how organizational structures, leadership support, and workflow design shape the development and sustainability of GI‐POCUS competence in critical care settings. Although this study was conducted in China, similar organizational and managerial challenges related to the implementation of POCUS among ICU nurses have been reported internationally, suggesting that the issues identified may reflect broader nursing management concerns rather than context‐specific barriers [19].

The demographic distribution of the participants revealed several notable patterns. Most respondents were bachelor‐educated nurses, reflecting the rising educational level among ICU nurses in China. Interestingly, male nurses accounted for 24.0% of GI‐POCUS users, substantially higher than the national average for male nurses [20], suggesting that traditional associations of technical‐intensive tasks with male roles may motivate greater adoption among male nurses. Among GI‐POCUS users, only 6.2% held senior professional titles and 9.1% were in managerial positions (e.g., head nurse), indicating that frontline clinical nurses with intermediate or primary professional titles, rather than management or teaching staff, constituted the primary user base. While senior and managerial nurses play key roles in institutional support and quality assurance, their focus on departmental management, quality control, and teaching reduces the opportunities for hands‐on GI‐POCUS practices [21]. These patterns suggest that GI‐POCUS implementation currently relies heavily on frontline clinical nurses, highlighting the need for nurse managers to proactively engage senior and supervisory nurses in competency governance, mentorship, and quality oversight rather than leaving skill development to individual initiative. Furthermore, the study participants had relatively long overall clinical and ICU‐specific work experience, indicating that nurses are more likely to acquire GI‐POCUS skills after reaching a certain level of professional experience. With accumulating experience, these nurses not only gain greater confidence in performing GI‐POCUS but also develop an enhanced appreciation of its value in patient care [22, 23].

At the departmental level, 81.6% of nurses reported that their departments permitted them to perform GI‐POCUS independently. This may be explained by the fact that most participants (88.1%) were from tertiary hospitals, which typically have superior equipment and technical expertise, placing them at the forefront of adopting advanced healthcare technologies. Nevertheless, the overall management systems for GI‐POCUS remain underdeveloped: 83.2% of the units lacked SOPs, 67.0% had no quality control mechanism, 69.7% lacked a mentorship program system, and 84.4% could not bill for GI‐POCUS examinations. This “permitted but unregulated” model represents a common managerial gap in the adoption of emerging nursing technologies, where clinical autonomy is granted without parallel investment in standardization, supervision, and accountability [24]. Without formal structures to guide practice, monitor quality, and support professional development, GI‐POCUS risks remaining an isolated technical activity rather than an integrated component of nursing care delivery.

Deficiencies in training, practice frequency, and analytical capability further highlight systemic challenges. More than half of the nurses performed GI‐POCUS without systematic training, only a minority demonstrated competence in qualitative and quantitative assessment or expressed confidence in diagnostic accuracy and sensitivity, and ultimately, just 14.8% rated their proficiency as “good.” These findings indicate that GI‐POCUS adoption in Chinese ICU nursing is at an early stage, constrained less by nurses’ motivation and more by the absence of structured training pathways, competency benchmarks, and managerial oversight [1, 25]. From a nursing management standpoint, episodic or one‐time training initiatives may support initial exposure but are insufficient to foster advanced proficiency or long‐term skill retention [26]. International reviews have similarly emphasized that formalized training programs, mentorship, and access to equipment are common facilitators or barriers to POCUS adoption among nurses and nurse practitioners worldwide, highlighting that the training challenges identified in this study are not unique to the Chinese context but reflect broader global patterns [27].

Multivariate logistic regression analysis indicated that ICU nurses’ mastery of GI‐POCUS is influenced by multiple factors, including usage frequency, proficiency in probe operation, qualitative and quantitative analyses ability, diagnostic accuracy and sensitivity, training experience, billable GI‐POCUS examinations, and documentation of GI‐POCUS findings in nursing charts.

Usage frequency was a significant predictor of proficiency (*p* < 0.001). Nurses with a higher procedural frequency demonstrated greater proficiency, indicating a positive correlation between hands‐on experience and skill competence. Frequent practice not only enhances probe manipulation and image interpretation but also allows nurses to accumulate judgment experience across diverse patients and clinical scenarios, thereby boosting diagnostic confidence. This finding reinforces the critical role of nurse managers in embedding GI‐POCUS into routine assessments and daily workflows [28]. As Su et al. reported [6], a higher clinical application frequency facilitates the transition from mechanical execution to integrated clinical decision‐making, highlighting the need for staffing models that intentionally create opportunities for repeated, supervised practice.

Probe handling skills, qualitative and quantitative analyses ability, and diagnostic accuracy and sensitivity were all closely associated with nurses’ GI‐POCUS mastery. Nurses lacking probe proficiency were significantly more likely to self‐assess themselves as “poor” (*p* < 0.05). Probe handling is foundational for GI‐POCUS, directly affecting image quality and anatomical clarity, and serving as a prerequisite for subsequent functional assessment [29]. Qualitative and quantitative analyses involve the comprehensive interpretation of parameters such as gastric motility, residual volume, and intestinal diameter changes, representing a critical step in translating imaging data into clinical decision‐making [30, 31]. A multivariate analysis showed that this ability significantly distinguished between “general” and “poor” proficiency (*p* < 0.001), but not between “good” and “poor,” suggesting that basic interpretative skills enable functional assessment but are insufficient for high‐level diagnostic judgment. Diagnostic perceived accuracy primarily reflects recognition of typical imaging patterns, significantly differentiating “general” from “poor” proficiency (*p* < 0.05), while perceived diagnostic sensitivity distinguished “good” from “poor” (*p* = 0.005), reflecting the ability to detect subtle or atypical abnormalities and provide early clinical alerts—key indicators of advanced GI‐POCUS proficiency [32]. These findings support a staged GI‐POCUS training pathway: foundational training emphasizing probe manipulation and high‐quality imaging, intermediate training reinforcing the quantitative analysis and standardized interpretation, and advanced training focusing on complex image recognition and integrative judgment. Continuous case review and image feedback can facilitate the skill progression from “poor” to “general” to “good,” promoting the transition from technical executor to clinical evaluator and advancing standardized, precise GI‐POCUS practice in critical care.

Training significantly facilitated nurses’ progression from “poor” to “general” mastery (*p* < 0.001) but did not significantly impact advancement from “general” to “good,” indicating that training plays a critical role during early skill acquisition, while proficiency depends on sustained clinical practice and experience accumulation [33]. Moreover, nurses in departments that incorporated GI‐POCUS into the billing system demonstrated significantly higher mastery than those in nonbilling units (*p* < 0.05), indicating that clear value identification and reward mechanisms could enhance nurses’ autonomous learning motivation and continuous practice willingness [34, 35]. From a management standpoint, formal recognition and reimbursement mechanisms may function as institutional signals that legitimize nursing‐led GI‐POCUS and encourage sustained engagement.

Finally, nurses who consistently documented GI‐POCUS findings in their nursing records demonstrated significantly higher mastery (*p* < 0.001). Continuous documentation and feedback enhance nurses’ POCUS competence and integration into routine clinical practice [17]. Hospitals should therefore embed ultrasound records into nursing information systems and establish standardized protocols and quality control mechanisms. Such measures transform documentation from a technical task into a managerial strategy that promotes accountability and reflection, thereby evolving GI‐POCUS from an individual practice into a sustainable organizational asset. This highlights documentation not merely as a technical task but as a managerial strategy to promote accountability, reflection, and integration into routine care.

This study revealed that GI‐POCUS competency is inherently domain‐specific. While ICU nurses demonstrated a relatively high ability to identify common GI dysfunctions frequently encountered in routine care, such as delayed gastric emptying (79.4%) and gastric distension or abnormal gastric volume changes (59.3%), the recognition rate for complex pathologies such as intestinal ischemia or ischemia was markedly lower (24.5%). This discrepancy may be attributed to the fact that the former is more frequently encountered in clinical practice and is closely related to routine nursing procedures, whereas the latter requires advanced image interpretation skills and a solid foundation in GI pathophysiology [36]. Additionally, the lack of systematic image recognition training and limited clinical practice opportunities may further restrict nurses’ abilities to identify complex GI pathologies. To enhance recognition accuracy, it is recommended that training programs incorporate case‐based image libraries [2] and multidisciplinary discussions to strengthen the understanding and interpretation of complex GI abnormalities.

The distribution of GI‐POCUS applications showed a clear stratification pattern. Nurses mainly used this technique for practical and frequently performed tasks such as assessing gastric emptying or residual volume (61.3%) and confirming feeding tube placement at bedside (53.2%) to guide enteral nutrition, reflecting its instrumental role in routine nursing workflows [13]. In contrast, applications requiring higher‐level interpretation and clinical decision‐making, such as screening for intestinal obstruction (22.1%) or evaluating intestinal mucosal perfusion and injury (14.2%), were less frequent, suggesting barriers in translating image acquisition into diagnostic reasoning. The contributing factors may include training programs focusing primarily on basic operations rather than image interpretation, unclear role boundaries for nurses in performing advanced GI‐POCUS assessments, and high workload pressures that favor time‐efficient tasks. Future training should, therefore, emphasize image interpretation and case‐based reasoning to facilitate the transition from technical performance to clinical judgment. Institutional support is also needed to clarify nurses’ scope of practice and integrate GI‐POCUS into routine rounds or nutritional assessments to promote sustainable use.

More than half of the ICU nurses could identify gastric structures and volumes in the transverse and longitudinal sections (51.4%), indicating a basic understanding of GI anatomy (Figure 3). However, the recognition of the pathological findings, such as intestinal tube distension (29.7%), intestinal wall thickening (23.2%), and gas‐fluid levels (26.9%), remained below 30%, with bowel wall perfusion signals showing the lowest accuracy (18.4%). This pattern mirrors previous findings in lung ultrasound studies, where nurses demonstrated proficiency in identifying basic features but struggled with complex pathological signs [37]. This may be due to limited exposure to advanced cases and insufficient training in complex image interpretation. Incorporating modules for pathological image recognition, establishing mentorship programs led by experienced ultrasound practitioners [19], and adopting AI‐assisted diagnostic tools may enhance nurses’ competence and confidence in interpreting complex GI sonographic findings.

Studies in technology‐intensive healthcare contexts have shown that nurses’ acceptance and sustained use of clinical innovations depend heavily on supportive management, tailored training strategies, and leadership engagement, rather than on individual technical skills alone [24]. Overall, the findings of this study suggest that GI‐POCUS proficiency among ICU nurses should be understood as an organizational capability shaped by leadership engagement, training governance, and workflow integration, rather than as an isolated technical skill. Addressing the observed competency gaps therefore requires coordinated managerial strategies that align education, practice opportunities, documentation systems, and institutional support to promote sustainable and safe GI‐POCUS implementation in critical care nursing.

### 4.1. Limitations

The limitations of this study are as follows. First, the cross‐sectional design established associations but could not infer causality between the identified factors and GI‐POCUS proficiency. Second, the use of convenience sampling, although yielding a large national sample, may have introduced selection bias, as participants were likely more interested or engaged in GI‐POCUS than the general ICU nurse population, potentially leading to an overestimation of proficiency and implementation rates. Third, although the study included a small number of nurses from pediatric and neonatal ICUs (*n* = 50, 2.5%), the sample size was insufficient to perform a separate analysis. Given the distinct anatomical and physiological characteristics of the GI tract in neonates and infants, and the greater technical difficulty of GI‐POCUS in this population, our findings may not be fully applicable to these specialized settings. Future studies should focus specifically on pediatric and neonatal ICU nurses to better understand their proficiency and training needs. Fourth, the data, including proficiency levels, practices, perceived accuracy, and sensitivity, were based on self‐reports via an online questionnaire, which is susceptible to social desirability and recall bias. The absence of objective skills assessment (e.g., via standardized tests or direct observation) means that actual clinical competency may differ from self‐rated proficiency. Finally, the study was conducted exclusively in China, and the findings, particularly regarding influencing factors such as billing policies and institutional support, may not be directly generalizable to healthcare systems in other countries with different training paradigms and resource allocation. Future research should incorporate longitudinal designs, objective competency assessments, and internationally validated instruments to further explore the dynamics of GI‐POCUS‐related skill acquisition and application.

## 5. Conclusions

The overall mastery of GI‐POCUS among ICU nurses in China is low. Usage frequency, probe operation proficiency, qualitative and quantitative analyses ability, diagnostic accuracy and sensitivity, training experience, and fees for GI‐POCUS examination are factors influencing proficiency. It is recommended to develop targeted, structured training programs, integrate GI‐POCUS into nursing workflow and electronic medical records, and implement supportive billing policies to improve the practical skills of ICU nurses in GI‐POCUS to provide higher‐quality and safer nursing services for patients who are critically ill.

## 6. Implications for Nursing Management

The findings of this study highlight GI‐POCUS as a nurse‐led, domain‐specific practice that requires active nursing leadership rather than reliance on individual skill acquisition alone. For nursing managers, GI‐POCUS competency should be understood as an organizational capability shaped by training governance, workflow integration, and institutional support. First, nurse leaders should develop structured and tiered training pathways aligned with the specific GI‐POCUS domains. Foundational training should prioritize probe operation and basic image acquisition to support routine applications such as gastric emptying and gastric residual volume assessment. Intermediate and advanced training should emphasize standardized image interpretation, quantitative analysis, and recognition of complex GI pathologies, particularly bowel perfusion abnormalities, through case‐based learning and mentorship. Second, GI‐POCUS should be embedded into routine ICU nursing workflows, including daily assessments and enteral nutrition management, to increase usage frequency and promote skill retention. Providing protected training time and adequate access to ultrasound equipment is essential to support sustained practice. Third, nursing leadership should establish organizational governance mechanisms, including SOPs, quality control systems, and documentation of GI‐POCUS findings within nursing records. Aligning GI‐POCUS practice with institutional recognition mechanisms, such as performance evaluation or billing policies where applicable, may further enhance its legitimacy and sustainability. Collectively, these leadership‐driven strategies can facilitate the safe, consistent, and sustainable integration of GI‐POCUS into critical care nursing practice.

## Author Contributions

Gang Wang, Yingying Liu, and Lili Wei were responsible for study design. Silong Gao, Hui Lin, Qian Wu, Debin Huang, Yueshuai Pan, Yuxiao Zhao, and Huimin Wei were responsible for data collection. Yingying Liu was responsible for data analysis. Gang Wang, Yingying Liu, and Lili Wei were responsible for manuscript writing and revisions.

## Funding

No funding was received for this manuscript.

## Disclosure

Gang Wang and Yingying Liu are the co‐first authors.

## Ethics Statement

This study was approved by the Committee on Medical Ethics of the Affiliated Hospital of Qingdao University (No. QYFYWZLL30728).

## Conflicts of Interest

The authors declare no conflicts of interest.

## Supporting Information

Additional supporting information can be found online in the Supporting Information section.

## Supporting information


**Supporting Information** The complete English version of the questionnaire used in this study is provided as Supporting File 1. English Version of the Questionnaire.

## Data Availability

The data that support the findings of this study are available on request from the corresponding author. The data are not publicly available due to privacy or ethical restrictions.

## References

[1] Yang L. , Lei L. , Zhang S. , Zhang X. , and Xu M. , Knowledge, Attitudes and Practices of Intensive Care Unit Nurses Regarding Critical Care Ultrasound: A Cross-Sectional Study in Southwestern China, Nursing in Critical Care. (2025) 30, no. 5, 10.1111/nicc.70170. PMC12424015 40931909

[2] Knutsen K. and Solbakken R. , Utilisation of Point-of-Care Ultrasound by Critical Care Nurses: A Scoping Review Protocol, BMJ Open. (2025) 15, no. 6, 10.1136/bmjopen-2025-100911. PMC12161366 40499962

[3] Millington S. J. , Narasimhan M. , Mayo P. H. , and Vieillard-Baron A. , Ten Influential Point-of-Care Ultrasound Papers: 2023 in Review, Journal of Intensive Care Medicine. (2025) 40, no. 6, 583–587, 10.1177/08850666241233556. 38374613 PMC12095873

[4] Boling B. and Solis A. , Point-of-Care Ultrasonography in the Critical Care Setting: Abdominal POCUS, AACN Advanced Critical Care. (2023) 34, no. 3, 216–227, 10.4037/aacnacc2023298. 37644636

[5] Watkins L. A. , Dial S. P. , Koenig S. J. , Kurepa D. N. , and Mayo P. H. , The Utility of Point-of-Care Ultrasound in the Pediatric Intensive Care Unit, Journal of Intensive Care Medicine. (2022) 37, no. 8, 1029–1036, 10.1177/08850666211047824. 34632837

[6] Su H. , Wang N. , Li Z. , Dong X. , Chen G. , and Cao L. , The Experiences of Critical Care Nurses Implementing Point-of-Care Ultrasound: A Qualitative Study, Nursing in Critical Care. (2025) 30, no. 4, 10.1111/nicc.70104. 40545829

[7] Morotti E. , Rovesti S. , Diambri C. et al., Development and Implementation of an Ultrasound Wireless Technology Educational Program for Nursing Students: A Quality Improvement Project, Nurs Rep. (2025) 15, no. 2, 10.3390/nursrep15020063. PMC11857979 39997799

[8] Al-Absi D. T. , Simsekler M. C. E. , Omar M. A. et al., Evaluation of Point-of-Care Ultrasound Training Among Healthcare Providers: A Pilot Study, Ultrasound Journal. (2024) 16, no. 1, 10.1186/s13089-023-00350-5. PMC10881927 38383673

[9] El Khoury D. , Pardo E. , Cambriel A. et al., Gastric Cross-Sectional Area to Predict Gastric Intolerance in Critically Ill Patients: The Sono-ICU Prospective Observational Bicenter Study, Critical Care Explorations. (2023) 5, no. 3, 10.1097/cce.0000000000000882. PMC10030198 36960310

[10] Reintam Blaser A. , Malbrain M. L. , Starkopf J. et al., Gastrointestinal Function in Intensive Care Patients: Terminology, Definitions and Management. Recommendations of the ESICM Working Group on Abdominal Problems, Intensive Care Medicine. (2012) 38, no. 3, 384–394, 10.1007/s00134-011-2459-y, 2-s2.0-84862539480. 22310869 PMC3286505

[11] Reintam Blaser A. , Preiser J. C. , Fruhwald S. et al., Gastrointestinal Dysfunction in the Critically Ill: A Systematic Scoping Review and Research Agenda Proposed by the Section of Metabolism, Endocrinology and Nutrition of the European Society of Intensive Care Medicine, Critical Care. (2020) 24, no. 1, 10.1186/s13054-020-02889-4. PMC7226709 32414423

[12] Camilleri M. , Parkman H. P. , Shafi M. A. , Abell T. L. , and Gerson L. , Clinical Guideline: Management of Gastroparesis, American Journal of Gastroenterology. (2013) 108, no. 1, 18–37, 10.1038/ajg.2012.373, 2-s2.0-84872013597. 23147521 PMC3722580

[13] Mihnovits V. , Reintam Blaser A. , Gualdi T. , Forbes A. , and Piton G. , Gastrointestinal Ultrasound in the Critically Ill: A Narrative Review and a Proposal for a Protocol, JPEN-Journal of Parenteral and Enteral Nutrition. (2024) 48, no. 8, 895–905, 10.1002/jpen.2687. 39403863

[14] Shankar N. , Kuo L. , Krugliak Cleveland N. et al., Point-of-Care Ultrasound in Gastroenterology and Hepatology, Clinical Gastroenterology and Hepatology. (2025) 23, no. 8, 1277–1290, 10.1016/j.cgh.2024.09.040. 39793722

[15] Brotfain E. , Erblat A. , Luft P. et al., Nurse-Performed Ultrasound Assessment of Gastric Residual Volume and Enteral Nasogastric Tube Placement in the General Intensive Care Unit, Intensive and Critical Care Nursing. (2022) 69, 10.1016/j.iccn.2021.103183. 34924254

[16] Neves R. P. S. , Assis A. P. , and Russo C. M. C. , Monitoring Enteral Feeding Tolerance in a Critically Ill Patient Using Point-of-Care Ultrasound, Revista Brasileira de Enfermagem. (2025) 78, no. 6, 10.1590/0034-7167-2025-0033. PMC12707605 41370602

[17] Yamada T. , Ehara J. , Funakoshi H. , Endo K. , and Kitano Y. , Effectiveness of Point of Care Ultrasound (POCUS) Simulation Course and Skills Retention for Japanese Nurse Practitioners, BMC Nursing. (2023) 22, no. 1, 10.1186/s12912-023-01183-2. PMC9872333 36691022

[18] Guo X. , Li X. , Wang Y. et al., Status and Influencing Factors of Nurses’ Perception of Toxic Leadership Behavior: A Cross-Sectional Study, Journal of Nursing Management. (2023) 2023, 7711237–7711238, 10.1155/2023/7711237. 40225629 PMC11918994

[19] Yamada T. , Kimura T. , Shigetomi K. et al., Barriers to and Facilitators of Point-of-Care Ultrasound Utilization Among Physicians, Nurse Practitioners, and Nurses in Japan: A Comparative Study, Ultrasound Journal. (2025) 17, no. 1, 10.1186/s13089-025-00399-4. PMC11723860 39792301

[20] Deng J. , Wang P. , Tian X. , Li K. , Yang L. , and Ding S. , Turnover Intention and Its Influencing Factors Among Male Nurses in China: A National-Scale Descriptive Study, BMC Nursing. (2024) 23, no. 1, 10.1186/s12912-024-02501-y. PMC11566173 39543638

[21] Elbus L. M. S. , Mostafa M. G. , Mahmoud F. Z. , Shaban M. , and Mahmoud S. A. , Nurse Managers’ Managerial Innovation and It’s Relation to Proactivity Behavior and Locus of Control Among Intensive Care Nurses, BMC Nursing. (2024) 23, no. 1, 10.1186/s12912-024-02084-8. PMC11251221 39014395

[22] Gong C. , Shen Y. , Wang J. , Zhang P. , and Li Z. , Application of Ultrasound Simulation Training in Intensive Care Nursing Teaching, BMC Medical Education. (2025) 25, no. 1, 10.1186/s12909-025-06968-4. PMC12007273 40247261

[23] Sreedharan J. K. , Karthika M. , Alqahtani J. S. et al., Routine Application of Lung Ultrasonography in Respiratory Care: Knowledge, Perceptions, and Barriers to Instigate, Advances in Medical Education and Practice. (2022) 13, 1395–1406, 10.2147/amep.S389013. 36411749 PMC9675578

[24] Barchielli C. , Marullo C. , Bonciani M. , and Vainieri M. , Nurses and the Acceptance of Innovations in Technology-Intensive Contexts: The Need for Tailored Management Strategies, BMC Health Services Research. (2021) 21, no. 1, 10.1186/s12913-021-06628-5. PMC8253682 34215228

[25] Tsolaki V. , Theodorakopoulou M. , and Zakynthinos E. , Regional Barriers in POCUS Training, Critical Care. (2025) 29, no. 1, 10.1186/s13054-025-05394-8. PMC12038941 40296129

[26] Gimenes F. R. E. , Stabile A. M. , Bernardes R. M. et al., Advancing Digital Education Technologies by Empowering Nurses With Point-of-Care Ultrasound: Protocol for a Mixed Methods Study, JMIR Research Protocols. (2024) 13, 10.2196/58030. PMC11541147 39441654

[27] Resnyk J. and Weichold A. , Barriers to Learning and Performing Point-of-Care Ultrasound (POCUS): An Integrative Review, Journal of Professional Nursing. (2024) 54, 54–62, 10.1016/j.profnurs.2024.06.007. 39266108

[28] Kitson A. L. , Harvey G. , Gifford W. et al., How Nursing Leaders Promote Evidence-Based Practice Implementation at Point-of-Care: A Four-Country Exploratory Study, Journal of Advanced Nursing. (2021) 77, no. 5, 2447–2457, 10.1111/jan.14773. 33626205

[29] Gilja O. H. and Nylund K. , Point-of-Care Ultrasound of the Gastrointestinal Tract, Journal of Medical Ultrasound. (2023) 31, no. 1, 1–7, 10.4103/jmu.jmu_5_23. 37180631 PMC10173834

[30] Roukhomovsky M. , Zieleskiewicz L. , Diaz A. et al., Ultrasound Examination of the Antrum to Predict Gastric Content Volume in the Third Trimester of Pregnancy as Assessed by MRI: A Prospective Cohort Study, European Journal of Anaesthesiology. (2018) 35, no. 5, 379–389, 10.1097/eja.0000000000000749, 2-s2.0-85045148543. 29210844

[31] Van de Putte P. and Perlas A. , Ultrasound Assessment of Gastric Content and Volume, British Journal of Anaesthesia. (2014) 113, no. 1, 12–22, 10.1093/bja/aeu151, 2-s2.0-84904015355. 24893784

[32] Shokoohi H. , Mayes K. D. , Peksa G. D. et al., Multi-Center Analysis of Point-of-Care Ultrasound for Small Bowel Obstruction: A Systematic Review and Individual Patient-Level Meta-Analysis, The American Journal of Emergency Medicine. (2023) 70, 144–150, 10.1016/j.ajem.2023.05.039. 37290251

[33] Schott C. K. , LoPresti C. M. , Boyd J. S. et al., Retention of Point-of-Care Ultrasound Skills Among Practicing Physicians: Findings of the VA National POCUS Training Program, American Journal of Medicine. (2021) 134, no. 3, 391–399.e8, 10.1016/j.amjmed.2020.08.008. 32931765

[34] Adhikari S. , Amini R. , Stolz L. et al., Implementation of a Novel Point-of-Care Ultrasound Billing and Reimbursement Program: Fiscal Impact, The American Journal of Emergency Medicine. (2014) 32, no. 6, 592–595, 10.1016/j.ajem.2014.02.051, 2-s2.0-84901671432. 24736125

[35] Hughes D. , Corrado M. M. , Mynatt I. et al., Billing I-AIM: A Novel Framework for Ultrasound Billing, Ultrasound Journal. (2020) 12, no. 1, 10.1186/s13089-020-0157-0. PMC7046859 32108277

[36] Olson M. C. , Bach C. R. , Wells M. L. et al., Imaging of Bowel Ischemia: An Update, from the AJR Special Series on Emergency Radiology, American Journal of Roentgenology. (2023) 220, no. 2, 173–185, 10.2214/ajr.22.28140. 35946859

[37] Zhao M. , Sun J. , Lou H. et al., Current Status and Influencing Factors of Lung Ultrasound Application Among ICU Nurses in China, Chinese Journal of Continuing Nursing Education. (2025) 40, no. 16, 1724–1731, 10.16821/j.cnki.hsjx.2025.16.008.

