# Supplementary material for: Factors Influencing ICU Nurses’ Proficiency in Point‐of‐Care Gastrointestinal Ultrasound: A National Cross‐Sectional Study
Source: J Nurs Manag. 2026 May 9;2026:3178958. doi: 10.1155/jonm/3178958 (PMC13157328; doi:10.1155/jonm/3178958)
Supplement: Supplementary file 1 — Supporting Information The complete English version of the questionnaire used in this study is provided as Supporting File 1. English Version of the Questionnaire. [file JONM-2026-3178958-s001.docx]

**Survey Questionnaire on the Proficiency and Current Application Status of Gastrointestinal Ultrasound Among ICU Nurses**

Dear Nursing Colleagues,

Greetings!

Point-of-care bedside ultrasound serves as a vital diagnostic and monitoring tool. In clinical nursing practice, it enables accurate and timely assessment of patient condition changes, guides clinical work, and enhances the quality of clinical care. This questionnaire aims to investigate ICU nurses' perceived proficiency and current application of gastrointestinal point-of-care ultrasound (GI-POCUS). There are no right or wrong answers. Please respond truthfully. All information is strictly confidential and will be used solely for academic research purposes. The survey takes approximately 10–15 minutes to complete. We kindly request you to complete it based on your actual experience. Thank you for your cooperation.

**Part I: Demographic Information**

1. Age: years old

2. Gender: □Male □Female

3. Education: □Junior college □Undergraduate □Masters or above

4. Professional title: □Primary level □Intermediate level □Advanced level

5. Job title: □No □Head Nurse/Director

6. Years of Work Experience: years

7. Years of ICU Experience: years

8. Hospital Grade: □Secondary □Tertiary

9. Categories of ICU: □General □Medical □Surgical □Emergency/Trauma

□Pediatric/Neonatal □Other

**Part II: Department Information**

1. Nurse-patient ratio：□Below 1:2 □1：2 to 1：3 □More than 1:3

2. Whether nurses were allowed to perform GI-POCUS independently: □No □Yes

3. Whether to incorporate GI-POCUS into routine daily assessments: □No □Yes

4. Whether GI-POCUS examinations were billable: □No □Yes

5. Whether to establish a standardized operating procedure for GI-POCUS: □No □Yes

6. Whether to establish a GI-POCUS quality control system: □No □Yes

7. Whether to establish a GI-POCUS mentorship system: □No □Yes

8. Number of ultrasound equipment in the department: units

**Part III: GI-POCUS training, practice, and skills**

1. Whether you have received GI-PCOUS training：□No □Yes

2. Duration of GI-POCUS use: □＜3 months □3 months–2 years □＞2 years
3. Frequency of GI-POCUS use: □Low □Relatively low □High
4. Whether nursing care plans are adjusted based on GI-POCUS result: □No □Yes

5. Acceptance of GI-POCUS–based nursing plan adjustments by attending physicians:

□Often □Occasionally □Rarely/Not at all

6. Whether GI-POCUS results are recorded in nursing charts: □No □Yes

7. Proficiency in probe operation: □No □Yes

8. Whether qualitative and quantitative analyses can be performed: □No □Yes

9. Perceived diagnostic accuracy of GI-POCUS: □Good □General □Poor

10. Perceived diagnostic sensitivity of GI-POCUS: □Good □General □Poor

**Part IV: GI-POCUS Proficiency Level**

1. Perceived Proficiency Level (Self-assessment, single choice):

□Good: Demonstrates solid theoretical knowledge and practical skills in GI-POCUS; independently performs procedures such as gastric residual volume monitoring, gastric motility assessment, and feeding tube placement verification with sound clinical judgment; and participates in teaching, training, or research activities. □Moderate: Possesses basic theoretical knowledge and operational skills in GI-POCUS; can perform certain tasks under guidance but has limited ability to handle complex cases or make comprehensive clinical judgments.

□Poor: Has a limited understanding of the basic concepts, principles, and operational techniques of GI-POCUS, with minimal independent practical experience.

**Part V： The clinical application of GI-POCUS**

1. Which of the following gastrointestinal dysfunctions can you identify using GI-POCUS? (Multiple selections permitted)

□Delayed gastric emptying

□Gastric distension

□Reduced or absent intestinal peristalsis

□Intestinal obstruction

□Abnormal gas–fluid levels in the intestine

□Intestinal dilation

□Intestinal necrosis / ischemia

□Ascites

2. In which of the following clinical scenarios have you used GI-POCUS? (Multiple selections permitted)

□Assess gastric emptying and residual volume

□Evaluate gastric contents characteristics

□Guide and confirm feeding tube position

□Determine enteral nutrition tolerance

□Assess intestinal motility

□Screen for intestinal obstruction risk

□Monitor ascites

□Assess intestinal mucosal perfusion and injury

3. Which of the following gastrointestinal ultrasound images can you accurately interpret? (Multiple selections permitted)

□Gastric transverse and sagittal sections

□Gastric wall thickness

□Intestinal peristaltic waves

□Intestinal tube distension

□Intestinal wall thickness

□Air-fluid levels in the intestines

□Visible Position of Feeding Tube

□Intestinal wall blood flow signals
